# Heterogeneous dermatitis complaints after change in drinking water treatment: a case report

**DOI:** 10.1186/1476-069X-5-18

**Published:** 2006-06-09

**Authors:** June M Weintraub, Magdalena Berger, Rajiv Bhatia

**Affiliations:** 1 Occupational and Environmental Health Section, San Francisco Department of Public Health, 1390 Market Street, San Francisco, CA 94102, USA

## Abstract

**Background:**

The disinfectant monochloramine minimizes the formation of potentially hazardous and regulated byproducts, and many drinking water utilities are shifting to its use.

**Case presentation:**

After a drinking water utility serving 2.4 million people switched to monochloramine for residual disinfection, a small number of residents complained of dermatitis reactions. We interviewed 17 people about their symptoms. Skin appearance, symptoms, and exposures were heterogeneous. Five respondents had history of hives or rash that preceded the switch to monochloramine.

**Conclusion:**

The complaints described were heterogeneous, and many of the respondents had underlying or preexisting conditions that would offer plausible alternative explanations for their symptoms. We did not recommend further study of these complaints.

## Background

Disinfection of public water supplies protects public health by inactivating microbial pathogens. Byproducts of disinfection with chlorine have been associated with bladder and rectal cancers and to adverse reproductive outcomes [1,2]. Because the disinfectant monochloramine minimizes the formation of potentially hazardous and regulated byproducts, many drinking water utilities are shifting to its use [3].

In February 2004 a water utility serving 2.4 million people in northern California replaced chlorine with monochloramine for secondary disinfection. Subsequently a small number of water customers raised concerns about skin rashes, attributing these rashes to the change in disinfection method. Skin complaints associated with water are not uncommon [4,5]. We are not aware of any previous work investigating this type of reaction as a specific response to the presence of monochloramine in the water supply. Dermatitis relating to water treatment is reported in two studies; one used a broad case definition [6], and the other revealed that the perception of change in water treatment was principally responsible for the symptoms, rather than any actual change in the water treatment [7]. Neither was specific to monochloramine.

In this context, we identified several possible explanations for the skin complaints that we received, including the following: (1) the symptoms were the result of underlying or preexisting conditions; (2) patients developed aquagenic pruritis or aquagenic pruritus of the elderly [4] independent of the change in water treatment and patients reported their symptoms knowing about a reported change in water treatment; and/or (3) reported symptoms were indeed the result of the change in water treatment. In order to gain insight into these hypotheses and evaluate the need for an epidemiologic investigation, local public health departments cooperated to develop a questionnaire to assess the homogeneity of the complaints. We hypothesized that homogeneity among the complaints might provide justification for a cross-sectional study of water customers; alternatively if we could not identify homogeneity, this might indicate the lack of a common cause, reducing the pressure for further investigation.

The questionnaire was administered between September 2004 and January 31 2005 to individuals who initiated calls to the health department. The public was made aware of the availability of the questionnaire through media reports and community meetings.

## Case presentation

Seventeen respondents called the health department and were administered the questionnaire by telephone. The average age was 65 (range 45–87). Fourteen respondents were female. Almost half were retired or disabled (n = 8). Eight respondents lived alone; nine had two or more people living in their households.

Ten respondents said their skin problems started in February 2004, five reported an onset date of March 2004, and two reported an onset date later than April 2004. Itchiness was reported by 15 respondents. Symptoms reported included dry skin (n = 8), bumps on the skin (n = 7), burning skin (n = 7), and red skin (n = 6). Four or fewer respondents each reported hives or welts, soreness, rash, flaky skin, pins and needles or tingling sensations and purple bumps. Most respondents reported the skin problem was on the arms and legs (n = 11) and torso (n = 10); four or fewer reported the problem was on the head, eyelids, shoulders, fingers, toes, or "all over".

Seven respondents had no previous skin problems other than poison ivy, poison oak, or acne. The remaining respondents reported history of hives or rash (n = 5), shingles, eczema, cracking of skin, skin cancer, psoriasis, burning sensations, lice or scabies (three or fewer respondents each). Thirteen respondents indicated that their problems were ongoing and eight felt that they were worse after contact with water. Two respondents had taken time off from work for doctor appointments as a result of the skin problem. A total of fourteen respondents had visited their doctor because of their skin problem, none remembered being given a diagnosis. Most respondents showered at least every other day (n = 11), and had previous allergies (n = 11). There were no common (n > 3) exposures to specific brands of cosmetics, body/bath products, laundry products, or medications.

## Conclusion

Our investigation indicates that the reported complaints were heterogeneous. Many of the respondents had underlying or preexisting conditions offering an alternative plausible explanation for their symptoms. Overall, results did not support the need for a wider study.

Our investigation was subject to several biases, and our findings should be interpreted with caution. The respondents were a convenience sample, and none were examined by a dermatologist as part of the investigation. The questionnaire was not validated. Most importantly, the investigation, the complaints, and speculation that these types of symptoms might be related to the change in water treatment were widely publicized in the local media.

Even with the widespread publicity, only 17 people volunteered to participate in the questionnaire in the five month period that it was open. Including seven who completed the questionnaire, a total of 48 calls from citizens with questions or complaints about chloramine were received by our health department between April 2004 and March 2006. Thirty-six of these callers were from outside of our health department jurisdiction, but within the water supply service area. The total population in the service area is 2.4 million.

This case investigation was designed to explore the complaints received by the health department. Although we recognized that the approach would not be sufficient to establish or disprove a causal relationship between the skin complaints and the presence of monochloramine in the water, we believe that our investigation was an appropriate step to determine the need for further investigation of these relationships. Nonetheless, clinicians working with populations served by utilities that are switching to monochloramine should be aware of our findings so that they are able to appropriately assess the timing, nature and alternative explanations for the symptoms.

Worldwide, many public drinking water providers are shifting to the use of monochloramine. In California, approximately 58% of the population in the 25 largest cities received water disinfected with monochloramine in 2005 [8]. Monochloramine is an effective disinfectant that minimizes the formation of trihalomethanes, for which there is strong evidence of a relationship with adverse health effects. We do not believe that the current evidence about the potential association between skin complaints and the presence of monochloramine in the water is a compelling reason to reconsider the use of monochloramine for residual water disinfection.

## Competing interests

All authors are paid employees of the City and County of San Francisco Department of Public Health. Ms. Berger's and Dr. Weintraub's positions are funded under a work order from the San Francisco Public Utilities Commission, the agency that provides the drinking water discussed in this case report. However, the San Francisco Public Utilities Commission did not have any role in the design and conduct of the investigation, in the collection, analysis, and interpretation of the data, nor in the preparation, review, or approval of the manuscript.

## Authors' contributions

JMW conceived the investigation, designed the questionnaire, participated in the data analysis, drafted parts of the manuscript, and critically revised the entire manuscript. MB designed and administered the questionnaire, performed the statistical analysis, drafted parts of the manuscript, and critically revised the entire manuscript. RB participated in the design of the investigation and questionnaire, and critically revised the manuscript. All authors read and approved the final manuscript.
